# Gynecomastia in Patients with Prostate Cancer: A Systematic Review

**DOI:** 10.1371/journal.pone.0136094

**Published:** 2015-08-26

**Authors:** Anders Fagerlund, Luigi Cormio, Lina Palangi, Richard Lewin, Fabio Santanelli di Pompeo, Anna Elander, Gennaro Selvaggi

**Affiliations:** 1 Department of Plastic Surgery, Institute of Clinical Sciences, Sahlgrenska Academy, University of Gothenburg, at Sahlgrenska University Hospital, Gothenburg, Sweden; 2 Department of Urology and Renal Transplantation, University of Foggia, Foggia, Italy; 3 Plastic Surgery Unit, Sant’Andrea Hospital, “Sapienza” University, Rome, Italy; Taipei Medical University, TAIWAN

## Abstract

**Introduction:**

Gynecomastia and/or mastodynia is a common medical problem in patients receiving antiandrogen (bicalutamide or flutamide) treatment for prostate cancer; up to 70% of these patients result to be affected; furthermore, this can jeopardise patients’ quality of life.

**Aims:**

To systematically review the quality of evidence of the current literature regarding treatment options for bicalutamide-induced gynecomastia, including efficacy, safety and patients’ quality of life.

**Methods:**

The PubMed, Medline, Scopus, The Cochrane Library and SveMed+ databases were systematically searched between January 1, 2000 and December 31, 2014. All searches were undertaken between January and February 2015. The search phrase used was:”gynecomastia AND treatment AND prostate cancer”. Two reviewers assessed 762 titles and abstracts identified. The search and review process was done in accordance with the PRISMA statement. The PICOS (patients, intervention, comparator, outcomes and study design) process was used to specify inclusion criteria. Quality of evidence was rated according to GRADE.

**Main Outcome Measures:**

Primary outcomes were: treatment effects, number of complications and side effects. Secondary outcome was: Quality of Life.

**Results:**

Eleven studies met the inclusion criteria and are analysed in this review. Five studies reported pharmacological intervention with tamoxifen and/or anastrozole, either as prophylactic or therapeutic treatment. Four studies reported radiotherapy as prophylactic and/or therapeutic treatment. Two studies compared pharmacological treatment to radiotherapy. Most of the studies were randomized with varying risk of bias. According to GRADE, quality of evidence was moderate to high.

**Conclusions:**

Bicalutamide-induced gynecomastia and/or mastodynia can effectively be managed by oral tamoxifen (10–20 mg daily) or radiotherapy without relevant side effects. Prophylaxis or therapeutic treatment with tamoxifen results to be more effective than radiotherapy.

## Introduction

Androgen deprivation therapy (ADT) is a therapeutic treatment recommended for asymptomatic patients with high risk, locally advanced (T3–4 or N+, M0) prostate cancer (PC). ADT is also recommended as a long-term adjuvant treatment, either isolated, or combined with external beam radiotherapy (EBRT), for node-positive (N+) patients. Surgical or medical castration, using luteinizing hormone-releasing hormone (LHRH) agonists, is recommended for metastatic (M1) prostate cancer. Antiandrogen treatment is recommended as a short-term treatment for patients receiving LHRH agonists. Bicalutamide is the most used nonsteroidal antiandrogen [1].

A very common side effect of antiandrogen treatment is the development of gynecomastia and/or mastodynia, sometimes referred to as bicalutamide-induced breast events (BEs) [1,2]. These side effects are due to an imbalance between estrogens and androgens in the breast tissue [3]. Stimulation of estrogen receptors in the breast tissue leads to growth; at the contrary, stimulation of androgen receptors inhibits this effect. Nonsteroidal antiandrogens block androgen receptors, which leads to increased secretion of luteinizing hormone (LH) by means of the feedback mechanism. Subsequently, increased LH stimulates testosterone secretion, which is then converted to estrogen by means of peripheral aromatization. The blockade of androgen receptors also increases the estrogens stimulatory effect on the breast tissue, as the androgen inhibitory effect is removed [4].

The cumulative prevalence of both these side effects can be as high as 70% [1,2]; this leads patients to discontinue antiandrogen treatment in 16.4% of cases [1]. According to various studies, in fact, gynecomastia can have a strong negative impact on patient’s Quality of Life (QoL). This is partly due to the loss of a male appearance [5,6]. Also, there have been reports of tamoxifen causing sexual impotence in male breast cancer patients, with negative effects on QoL [7].

Classifications of gynecomastia, as reported in literature, are based on: appearance of the breast, composition ratio between fat and glandular tissue, or a combination. In order to assist in the diagnosis process, ultrasound and mammography are often used [8].

Gynecomastia and mastodynia can be managed by pharmacological treatments, surgical, as well as by low-dose radiotherapy (RT) of the breast tissue.

Pharmacological methods aimed at preventing or treating gynecomastia include the antiestrogen tamoxifen and the aromatase inhibitor anastrozole [2,7,9–16]. Tamoxifen aims at reducing estrogens effect in breast tissue by blocking the estrogen receptors. Anastrozole aims at reducing the peripheral aromatization of androgens into estrogen, and thereby reducing the estrogen effect on breast tissue [4]. RT prevents gynecomastia by inducing hypoplasia or aplasia in the irradiated tissue. There is also a reduction in the number of ducts in irradiated breasts [14].

## Aims

To systematically review the quality of evidence of the current literature regarding treatment options for antiandrogen induced gynecomastia, including efficacy, safety and patients’ QoL.

## Methods

### Information sources

Studies were identified by searching systematically in the electronic databases PubMed, Medline, Scopus, The Cochrane Library and SveMed+. The search phrase used was: “gynecomastia AND treatment AND prostate cancer”. The search and review process was done in accordance with the PRISMA statement. Searches were restricted to articles in the English language published after 2000. All searches were undertaken between January and February 2015. No registered review protocol exists.

### Study selection and data collection process

We searched for, and assessed studies comparing different treatment regimes for antiandrogen associated gynecomastia. Pharmacological treatment, RT and surgery were accepted. Both prophylactic and therapeutic treatments were accepted. Studies to be included in this review had to match predetermined criteria according to the PICOS approach. Criteria for inclusion and exclusion are specified in Table 1. No limitations were applied on ethnicity, age of patients or stage of prostate cancer. Studies reporting gynecomastia with other etiology were excluded as they are reported in a separate study [8].

**Table 1:** PICOS criteria for inclusion and exclusion of studies.

Parameter	Inclusion criteria	Exclusion criteria
Patients	Patients of any age with prostate cancer/treatment associated gynecomastia	Other types of gynecomastia
Intervention	Pharmacological, surgical or radiotherapeutic treatments	
Comparator	How effective are the different treatment methods	
Outcomes	*Primary outcome measures*: Treatment results and number of complications/side effects. *Secondary outcome measures*: QoL	
Study design	Randomized controlled trials, non-randomized controlled trials, retrospective, prospective, or concurrent cohort studies. At least 20 patients. At least 1 year follow-up. Published in English.	Reviews, expert opinion, comments, letter to editor, case reports, studies on animals, conference reports. Less than 20 patients. Shorter follow-up than 1 year. Studies with no outcomes reported. Published before 2000. Published in any other language than English.

Two reviewers independently screened all search results, titles and abstracts, in order to find studies matching the predetermined inclusion criteria. Any apprehension was resolved by discussion. The data from included studies were extracted to an extraction form. Search process and reasons for exclusion are presented in a flow diagram (S1 Fig).

### Quality assessment strategy and risk of bias

The included studies have been assessed for bias in order to evaluate validity and risk for over- or under-estimating the true intervention effect. To determine the risk of bias, one reviewer assessed all the included studies by using the Cochrane Collaboration's 'Risk of bias' tool. This tool provides support for judging specific features of a study in a specific 'Risk of bias' table. In Table 2 each entry can be assessed as: 'low risk', 'high risk' or 'unclear risk'. 'Unclear risk' indicates lack of information in a study or uncertainty for risk of bias [17] (Table 2).

**Table 2:** Risk of bias according to The Cochrane Collaboration's 'Risk of bias' tool.

Publi-cation	Ran-dom sequ-ence genera-tion	Allo-cation conceal-ment	Blin-ding of partici-pants and personnel	Blin-ding of out-come assess-ment	Incom-plete out-come data	Selec-tive repor-ting	Other sources for bias	Sum-mary
Ser-retta et al. [2]	Unclear	Yes	Unclear	Unclear	Yes	Unclear	Co-authors employed by Astra-Zeneca	Unclear risk of bias
Bedog-netti et al. [9]	Yes	Yes	No	No	Yes	Unclear		High risk of bias
Fradet et al. [10]	Yes	Yes	Yes	Yes	Yes	Unclear	Funding by Astra-Zeneca	Low risk of bias
Saltz-stein et al. [4]	Yes	Yes	Yes	Yes	Yes	Unclear	Co-authors employed by Astra-Zeneca	Low risk of bias
Boc-cardo et al. [7]	Yes	Unclear	Yes	Yes	Yes	Unclear	Funding by Astra-Zeneca	Unclear risk of bias
Ozen et al. [11]	Unclear	Yes	No	No	Yes	Unclear		High risk of bias
Van Poppel et al. [12]	No	No	No	No	Yes	Unclear	Sponsored by Astra-Zeneca	High risk of bias
Tyrrell et al. [13]	Yes	Yes	Yes	Yes	Yes	Unclear	Funding by Astra-Zeneca. Co-authors employed by Astra-Zeneca	Low risk of bias
Wid-mark et al. [14]	Unclear	Unclear	No	No	Yes	Unclear		High risk of bias
Per-doná et al. [15] Di Loren-zo et al. [16]	Yes	Yes	No	No	Yes	Unclear		High risk of bias

The evidence across included studies was rated using the GRADE system. This system is designed for reviews and guidelines that examine different treatments or interventions. Using pre-specified criteria quality of evidence can be rated by assessing risk of bias (“internal validity”), directness and precision (“external validity”) [18]. The quality of evidence for each outcome measure was assessed to be: very low, low, moderate, or high (Table 3).

**Table 3:** GRADE analysis and definition.

Quality of evidence	Definition
High	We are very confident that the true effect lies close to that of the estimate of the effect
Moderate	We are moderately confident in the effect estimate: The true effect is likely to be close to the estimate of the effect, but there is a possibility that it is substantially different
Low	Our confidence in the effect estimate is limited: The true effect may be substantially different from the estimate of the effect
Very low	We have very little confidence in the effect estimate: The true effect is likely to be substantially different from the estimate of effect

### Main outcome measures

Primary outcomes were: treatment effects, number of complications/side effects. Secondary outcomes were: QoL.

## Results

A total of 762 titles and abstracts were obtained through the systematic literature search. After a preliminary screening, 24 duplicates and 694 studies that clearly did not match inclusion criteria were removed. 44 abstracts and full texts were examined in respect to inclusion criteria. 11 studies met inclusion criteria and were included in this review.

### Validity of included studies

The validity of the included studies is presented in Table 2.

### Main outcome measures

Bicalutamide (or flutamide) treatment is highly associated with gynecomastia and/or mastodynia; prevalence of these side effects can be as high as 70%. These are predictable side effects that can be prophylactically managed either by pharmacological treatment or RT.

If no prophylactic treatment has been initiated, therapeutic treatment with drugs or RT can be started on the onset of symptoms [2,7,9–16].

Seven studies included patients with T1 or T1b—T3–4, any N and M0 [4,10,11–13,15,16]. Three studies included patients with non-metastatic, local or locally advanced PC [2,7,14]. One study provided no information on staging but excluded metastatic PC [9].

Primary and secondary outcomes are summarized in Tables 4 and 5. Table 4 summarizes treatment effects, both pharmacological treatment with either tamoxifen and/or anastrozole, and RT. Side effects and complications are also presented in Table 4 [2,7,9–16]. Table 5 summarizes QoL following gynecomastia treatment [10,15,16].

**Table 4:** Primary outcomes.

Publication	Design	Intervention and results	Side effects
Serretta et al. [2]	Randomized, multicentre trial	Arm A: tamoxifen therapy reduced BEs to 28% of patients (*P* < 0.001). Arm B: tamoxifen prophylaxis gave significant reduction of BEs to 44% (*P* < 0.001).	Arm A: 2.4% discontinued tamoxifen and 3.6% discontinued bicalutamide. Arm B: 2.5% discontinued both treatments.
Bedognetti et al. [9]	Randomized, multicentre phase 3 trial	Arm A: daily tamoxifen prophylaxis resulted in 31.7% developing gynecomastia (*P* < 0.0001) and 12.2% mastalgia (*P* = 0.001). Arm B: tamoxifen weekly after 8 weeks of daily treatment resulted in 74.4% developing gynecomastia (*P* < 0.0001) and 46.1% mastalgia (*P* = 0.001).	Arm A: 24%. Arm B: 22%
Fradet et al. [10]	Randomized, double-blind, multicentre trial	Arm A: increased doses of tamoxifen daily decreased the number of BEs, compared to placebo, ranging from 86.2% in the 1 mg group to 8.8% in the 20 mg group (*P* ≤ 0.0002). Arm B: placebo, 96.7% experienced BEs.	Arm A: 19.8% withdrew. Arm B: 16.7% (bicalutamide monotherapy)
Saltzstein et al. [4]	Randomized, double-blind, multicentre trial	Arm A: tamoxifen prophylaxis patients were significantly (3.22 times) less likely to develop gynecomastia and/or mastodynia (relative risk estimate, 95% CI 1.28, 7.69). The difference between the anastrozole group and the placebo group was not significant (*P* = 0.749). Therapeutic treatment gave resolution of gynecomastia and/or mastodynia in 65.4% in the tamoxifen group and 18.8% in the anastrozole group. Arm B: placebo, 88.8% later received tamoxifen due to BEs. This treatment significantly reduced gynecomastia and/or mastodynia in 71.8%.	13.2% withdrew. 0.9% (one patient) withdrew due to increased serum AST and ALT levels.
Boccardo et al. [7]	Randomized, double-blind, multicentre trial	Arm A: 10% of the tamoxifen group and 51% of the anastrozole developed gynecomastia (*P* < 0.001); 6% of the tamoxifen group and 27% of the anastrozole group experienced mastodynia (*P* = 0.006). Arm B: control, 73% developed gynecomastia (*P* < 0.001) and 39% experienced mastodynia (*P* = 0.006).	Arm A: 35.1%% of the tamoxifen group and 69.5% of the anastrozole group. Arm B: 37.5%
Ozen et al. [11]	Randomized, multi-institutional trial	Arm A: prophylactic RT resulted in 15.8% (*P* < 0.001) developing gynecomastia and 36.4% mastodynia. Arm B: control, 50.8% (*P* < 0.001) developed gynecomastia and 49.2% breast pain.	Arm A: 11.3% (related to bicalutamide). Arm B: 11.1% (related to bicalutamide).
Van Poppel et al. [12]	Open label, multicentre study	Arm A: therapeutic RT resulted in 36.6% experienced improved or resolved gynecomastia and mastodynia; 22% had no change and 24.4% experienced worsening of symptoms.	Arm A: 43.9%. All RT related side effects were transient.
Tyrrell et al. [13]	Randomized, double-blind, multicentre trial	Arm A: prophylactic RT resulted in an incidence of gynecomastia between 50% and 52% (OR 0.13, 95% CI 0.04, 0.38, *P* <0.001). Arm B: sham RT resulted in an incidence of gynecomastia between 81% and 85% (OR 0.13, 95% CI 0.04, 0.38, *P* <0.001).	Arm A: 33% (RT related), all of which were short-lived. Arm B: 2% (RT related).
Widmark et al. [14]	Randomized, Scandinavian trial	Arm A: prophylactic RT resulted in an incidence of gynecomastia between 28% and 44%. Breast tenderness was reported in 43% of patients (*P* < 0.001). Arm B: control, incidence of gynecomastia between 71% and 78%. Breast tenderness reported in 75% of patients (*P* < 0.001).	Some patients reported skin irritation
Perdonà et al. [15] Di Lorenzo et al. [16]	Randomized, multicentre trial	Arm A: prophylactic RT resulted in 34% developing gynecomastia (OR 0.51, *P* = 0.008) and 30% mastodynia (OR 0.43, *P* = 0.02). Arm B: tamoxifen daily resulted in 8% developing gynecomastia (OR 0.1, 95% CI, *P* = 0.0009) and 6–7% breast pain (OR 0.1, *P* = 0.009). Arm C: control, 67–70% developed gynecomastia and 57–58% mastodynia. Tamoxifen later significantly reduced gynecomastia and mastodynia in arm C (OR 0.2, *P* = 0.02)	Arm A: highest number of side effects where 19–40% experienced short-lived skin irritations, rashes or nipple erythema.

**Table 5:** Secondary outcome.

Publication	Method	Intervention	Results	Conclusion
Perdonà et al. [15] Di Lorenzo et al. [16]	Validated questionnaire (EORTC QLQ-c30)	Arm A: prophylactic RT. Arm B: tamoxifen. Arm C: control	No differences in mean global health scores were found when comparing the two intervention groups.	QoL was not negatively affected by either treatment option.
Boccardo et al. [7]	Self-adminstered, validated questionnaire.	Arm A: tamoxifen or anastrozole. Arm B: control	No differences between groups concerning sexual interest. Minor difference related to sexual functioning, scores increased at 6-months for the anastrozole and control groups while the tamoxifen group remained unchanged. Data on other domains were not reported in detail.	No harmful effects on QoL were caused by the addition of tamoxifen or anastrozole to bicalutamide. Tamoxifen did not worsen sexual interest or functioning.
Fradet et al [10]	Patients were interviewed	Arm A: tamoxifen daily (1–20 mg). Arm B: placebo	Arm A: erectile dysfunction was lowest in the 20 mg group (2.9%) and highest in the 10 mg group (11.8%). Arm B: erectile dysfunction in 3.3%.	No major differences in erectile dysfunction between placebo and the different tamoxifen doses.
Saltzstein et al. [4]	Self-adminstered questionnaire	Arm A: tamoxifen or anastrozole daily.Arm B: placebo	Fewer than 5% in all treatment groups reported loss of libido or erectile difficulties.	No evidence of increased sexual dysfunction with either treatment.

### Drug therapy

The antiestrogen tamoxifen and the aromatase inhibitor anastrozole have both been used as pharmacological treatments, either as prophylactic or therapeutic regimens.

Prophylactic treatment with tamoxifen, 10–20 mg daily started simultaneously as bicalutamide treatment, is highly effective in preventing both gynecomastia and mastodynia [2,4,7,9,10]. Six studies investigated the use of tamoxifen as prophylactic treatment. All studies reported a reduction in BEs. Three studies reported a prevalence of BEs ranging from 8.8% to 44% following prophylactic treatment [2,9,10]. Saltzstein et al. reported that patients receiving tamoxifen were 3.22 times less likely to develop BEs [4]. Boccardo et al. [7] reported that prophylactic administration of tamoxifen reduced the risk of developing gynecomastia and/or mastodynia by seven and six times, respectively, when compared to a control group. Prophylactic treatment with tamoxifen was also reported to be safe and generally well tolerated [2,7,9]; again, Boccardo et al. [7] reported side effects in 35% of the studied population.

Prophylactic treatment with anastrozole conversely failed to provide similar benefits in reducing BEs. Saltzstein et al. [4] reported anastrozole to provide no advantage over placebo. Boccardo et al. [7] reported that anastrozole, compared to tamoxifen, was five times less efficient on gynecomastia and four times less efficient on mastodynia. Moreover, treatment with anastrozole resulted in twice as many side effects when compared to tamoxifen [7].

Two studies reported tamoxifen as therapeutic treatment. Saltzstein et al. [4] reported that 65.4% of patients who received therapeutic treatment with tamoxifen, 20 mg daily, experienced resolution of gynecomastia and/or mastodynia. In the same study the control group developed BEs, and therefore 88.8% of these patients received tamoxifen: resolution occurred in 71% of treated subjects [4]. Serretta et al. [2] compared therapeutic tamoxifen treatment with tamoxifen prophylaxis. Authors found a statistically significant difference where therapeutic treatment reduced the prevalence of BEs to 28%, while prophylaxis reduced the prevalence to 35% [2].

Taking these findings together, tamoxifen results to be more effective than anastrozole in both preventing and treating gynecomastia and mastodynia. Tamoxifen is most effective when administered daily; when switching to a weekly schedule, instead, the preventive effect declined [9]. There seems to be a dose-response relationship between the administered dose and the level of BEs. Fradet et al. reported that increased doses of tamoxifen, from 1 mg to 20 mg, decreased the number of BEs in a dose-respondent manner. There was no increase in adverse effects in the 20 mg group compared with lower doses or placebo [10]. This suggests that daily administered, prophylactic treatment with tamoxifen (10–20 mg) is an effective treatment, with an acceptable risk of minor side-effects [2,4,7,9,10,15].

### Radiotherapy

RT has been used either as prophylactic or therapeutic treatment for BEs [11–14]. Three studies reported on prophylactic RT. Two studies reduced gynecomastia to 28–50%, compared to control groups where 71–85% experienced gynecomastia [13,14]. Ozen et al. [11] reduced gynecomastia to 15.8%, compared to a control group with an incidence of 50%. Two of the studies reported a decrease of mastodynia to 36–43%, compared to control groups with an incidence of 49–75%. Compared to no treatment or sham RT, prophylactic RT significantly decreased the prevalence of gynecomastia to approximately one third of patients [11,13–16]. Nevertheless, RT was less effective than prophylactic tamoxifen, which in one study [8] reduced gynecomastia to approximately 8% [15– 16]. Side effects following prophylactic RT were present in one third of the patients, but these were usually mild and short-lived. Therapeutic RT results to be less effective than prophylactic RT. Van Poppel et al. [12] reported improvement or resolution of gynecomastia in 7–26% of patients, while mastodynia improved or resolved in 7–29%. Almost a quarter (21–24%) of patients actually experienced worsening of symptoms. Side effects were in the range of 40% and these tended to be mild and transient [12,15–16].

Taking these findings together, prophylactic RT results to be more effective than therapeutic RT, yet less effective than tamoxifen. The occurrence of RT related side effects is not negligible, but they are mild and transient.

### Quality of life

Boccardo et al. [7] reported no differences concerning sexual interest when comparing patients treated with tamoxifen or anastrozole. There was an increase in sexual functioning scores in the anastrozole and the control group at six months, while the tamoxifen group scores remained unchanged. Authors concluded that the addition of tamoxifen or anastrozole to bicalutamide was not causing harmful effects to QoL, and that tamoxifen did not worsen sexual interest or functioning. Perdonà et al. [15] and Di Lorenzo et al. [16] assessed QoL using the EORTC QLQ-c30 questionnaire, which scores physical, role, emotional, cognitive, social functions and overall health status. The questionnaire also contains a multi-item scale that scores individual physical symptoms. Authors could not show any differences in mean global health scores, when comparing tamoxifen to RT, thus concluding that no negative effect on QoL is caused by any of the two treatment approaches.

Fradet et al. [10] found no differences concerning erectile dysfunction when comparing different doses of tamoxifen and placebo. These results are strengthened by Saltzstein et al. [4] who reported no increase of sexual dysfunction from either tamoxifen or anastrozole. Both authors conclude that no negative effect on sexual functioning is to be expected from tamoxifen treatment for BEs.

Taking these findings together, no negative effects on QoL are to be expected from either tamoxifen or RT, when managing BEs.

## Discussion

Gynecomastia and/or mastodynia are very common medical problems in patients receiving nonsteroidal antiandrogen treatment for prostate cancer. Different treatments are currently in use. No systematic report on QoL following treatment for BEs is present in the literature.

In this review different treatments for nonsteroidal antiandrogen-induced gynecomastia and/or mastodynia have been evaluated: tamoxifen had a significant effect on BEs, both as prophylactic and as therapeutic treatment.

Studies comparing tamoxifen to treatment with the aromatase inhibitor anastrozole have been evaluated. Anastrozole failed to provide any significant improvement in treating antiandrogen-induced gynecomastia and/or mastodynia. When comparing prophylactic RT to tamoxifen, tamoxifen appears to be the most effective treatment; however, only one study compared these two treatments. The prophylactic treatment regime with daily administration of tamoxifen 10–20 mg is showing the best results.

Within the articles selected, the number of patients reported was irrelevant to the final assessment of the quality of evidence, since in these works the evidence was determined by internal and external validity and precision.

Five studies concerning pharmacological treatment have been included [2,4,7,9,10], according to The Cochrane Collaboration´s risk of bias tool: two have been classified as presenting “low risk of bias”, two have “unclear risk of bias”, and one study has “high risk of bias”. No description of the randomization process, blinding or allocation concealment is reasons for rating “unclear risk of bias”. Lack of blinding is the reason for “high risk of bias” in one study. The quality of evidence of the studies concerning pharmacological treatment, and included in this review was scored high (Table 3).

Four studies concerning RT have been included [11–14]: one has been classified as presenting “low risk of bias”, while three have “high risk of bias.” No randomization, insufficient description of randomization process, and no blinding, have been considered as factors to increase the risk of bias. According to GRADE, this is sufficient enough to affect the internal validity and thereby lead to serious study limitations, which leads to lower levels of evidence. The quality of evidence of the studies concerning RT, and included in this review was scored moderate (Table 3).

The study that compares RT to pharmacological treatment has been classified as presenting “high risk of bias” as there is no blinding.

Two studies evaluating QoL [7,15–16] following gynecomastia treatment have “unclear” and “high risk of bias”. One study has insufficient information on allocation concealment and the other one has no blinding. This affects the internal validity of the studies and is enough to downgrade the overall quality of evidence due to serious study limitations.

The quality of evidence of these studies concerning QoL following gynecomastia treatment, and included in this review, is scored moderate (Table 3). Both studies used validated questionnaires to assess QoL outcomes; however, the questionnaires used were different; subsequently, it is difficult to draw general conclusions from these results. Furthermore, QoL is reported only as a secondary outcome in both articles. Further studies on QoL are needed.

Two more studies reported on sexual functioning and erectile problems [4,10]. Both studies had “low risk of bias” and quality of evidence was scored high (Table 3).

Four of the five studies concerning pharmacological treatment [2,4,7,10], and two of the four concerning RT [12,13], received financial support, or include co-authors that are directly employed by the drug producing company (AstraZeneca). This represents, in fact, another possible source of bias that could affect the internal validity, but it was not given enough importance to impact the overall quality of evidence.

Future studies should be carefully designed in advance in order to minimize the risk of bias, and to subsequently achieve higher quality of evidence. Authors are recommended to use adequate allocation sequence generation and concealment, as well as blinding of participants, personnel and outcome assessors. All outcome data should be reported to avoid incomplete reporting. To avoid selective reporting, a protocol with pre-specified outcomes should be available. All methods concerning study characteristics must be reported in the article to avoid “unclear risk of bias” due to missing information.

This review has several limitations: it is limited to articles published in English only, and we found no study concerning surgical treatment for BEs that matched inclusion criteria, thus making it impossible to discuss surgical treatment.

## Conclusions

The result from this systematic review is that the quality of evidence regarding the management of BEs ranges from moderate to high.

Available evidence suggests that nonsteroidal induced gynecomastia and/or mastodynia can be safely and effectively prevented in clinical practice with prophylactic tamoxifen (10–20 mg daily) or RT. Tamoxifen can also be used as an effective therapeutic treatment. Tamoxifen appears to be more effective in preventing gynecomastia and/or mastodynia than RT. No negative effect on QoL and/or sexual functioning is to be expected from tamoxifen when used for antiandrogen-induced gynecomastia. Anastrozole is reported to be the least successful treatment, and should not be recommended either as prophylactic or therapeutic treatment in clinical practice for antiandrogen-induced gynecomastia. Future high quality studies with low risk of bias are needed to further evaluate the effect on QoL in this patient group.

## Supporting Information

S1 Fig Flow diagram—search process. The diagram depicts number of records identified, included and excluded as well as reasons for exclusion.
(EPS)

## References

[1] Heidenreich A, Bastian P J, Bellmunt J, Bolla M, Joniau S, van der Kwast T, et al EAU guidelines on prostate cancer. Part II: treatment of advanced, relapsing, and castration-resistant prostate cancer. European urology 2014;65(2):467–479. 10.1016/j.eururo.2013.11.002 24321502

[2] Serretta V, Altieri V, Morgia G, Nicolosi F, De Grande G, Mazza R, et al A randomized trial comparing tamoxifen therapy vs. tamoxifen prophylaxis in bicalutamide-induced gynecomastia. Clinical genitourinary cancer 2012;10(3):174–179. 10.1016/j.clgc.2012.03.002 22502790

[3] Braunstein G D. Aromatase and gynecomastia. Endocrine-related cancer 1999;6(2):315–324. 10731125 10.1677/erc.0.0060315

[4] Saltzstein D, Sieber P, Morris T, Gallo J. Prevention and management of bicalutamide-induced gynecomastia and breast pain: randomized endocrinologic and clinical studies with tamoxifen and anastrozole. Prostate cancer and prostatic diseases 2005;8(1):75–83. 15685254 10.1038/sj.pcan.4500782

[5] Davanço R A S, Neto M S, Garcia É B, Matsuoka P K, Huijsmans J P R, Ferreira L M. Quality of life in the surgical treatment of gynecomastia. Aesthetic plastic surgery 2009;33(4):514–517. 10.1007/s00266-008-9213-z 18953597

[6] Barros A C S D D, Sampaio M D C M. Gynecomastia: physiopathology, evaluation and treatment. Sao Paulo Medical Journal 2012;130(3):187–197. 22790552 10.1590/S1516-31802012000300009 PMC10876201

[7] Boccardo F, Rubagotti A, Battaglia M, Di Tonno P, Selvaggi F P, Conti G, et al Evaluation of tamoxifen and anastrozole in the prevention of gynecomastia and breast pain induced by bicalutamide monotherapy of prostate cancer. Journal of clinical oncology 2005;23(4):808–815. 15681525 10.1200/JCO.2005.12.013

[8] Fagerlund A, Lewin R, Rufolo G, Elander A, Santanelli di Pompeo F, Selvaggi G. Gynecomastia: a systematic review. Journal of Plastic Surgery and Hand Surgery 2015. In press. 10.3109/2000656X.2015.1053398 26051284

[9] Bedognetti D, Rubagotti A, Conti G, Francesca F, De Cobelli O, Canclini L, et al An open, randomised, multicentre, phase 3 trial comparing the efficacy of two tamoxifen schedules in preventing gynecomastia induced by bicalutamide monotherapy in prostate cancer patients. European urology 2010;57(2):238–245. 10.1016/j.eururo.2009.05.019 19481335

[10] Fradet Y, Egerdie B, Andersen M, Tammela T L, Nachabe M, Armstrong J, et al Tamoxifen as prophylaxis for prevention of gynaecomastia and breast pain associated with bicalutamide 150mg monotherapy in patients with prostate cancer: a randomised, placebo-controlled, dose–response study. European urology 2007;52(1):106–115. 17270340 10.1016/j.eururo.2007.01.031

[11] Ozen H, Akyol F, Toktas G, Eskicorapci S, Unluer E, Kuyumcuoglu U, et al Is prophylactic breast radiotherapy necessary in all patients with prostate cancer and gynecomastia and/or breast pain?. The Journal of urology 2010;184(2):519–524. 10.1016/j.juro.2010.03.137 20620411

[12] Van Poppel H, Tyrrell C J, Haustermans K, Van Cangh P, Keuppens F, Colombeau P, et al Efficacy and tolerability of radiotherapy as treatment for bicalutamide-induced gynaecomastia and breast pain in prostate cancer. European urology 2005;47(5):587–592. 15826748 10.1016/j.eururo.2004.12.003

[13] Tyrrell C. J, Payne H, Tammela T L, Bakke A, Lodding P, Goedhals L, et al Prophylactic breast irradiation with a single dose of electron beam radiotherapy (10 Gy) significantly reduces the incidence of bicalutamide-induced gynecomastia. International Journal of Radiation Oncology* Biology* Physics 2004;60(2):476–483. 10.1016/j.ijrobp.2004.03.022 15380582

[14] Widmark A, Fosså S D, Lundmo P, Damber J E, Vaage S, Damber L, et al Does prophylactic breast irradiation prevent antiandrogen-induced gynecomastia? Evaluation of 253 patients in the randomized Scandinavian trial SPCG-7/SFUO-3. Urology 2003;61(1):145–151. 12559286 10.1016/s0090-4295(02)02107-6

[15] Perdonà S, Autorino R, De Placido S, D'Armiento M, Gallo A, Damiano R, et al Efficacy of tamoxifen and radiotherapy for prevention and treatment of gynaecomastia and breast pain caused by bicalutamide in prostate cancer: a randomised controlled trial. The lancet oncology 2005;6(5):295–300. 15863377 10.1016/S1470-2045(05)70103-0

[16] Di Lorenzo G, PerdonÀ S, De Placido S, D'Armiento M, Gallo A, Damiano R, et al Gynecomastia and breast pain induced by adjuvant therapy with bicalutamide after radical prostatectomy in patients with prostate cancer: the role of tamoxifen and radiotherapy. The Journal of urology 2005;174(6):2197–2203. 16280763 10.1097/01.ju.0000181824.28382.5c

[17] Higgins J P, Green S. Cochrane handbook for systematic reviews of interventions. The Cochrane Library 2005;4(6).

[18] Group Gw. Grading quality of evidence and strength recommendations. 2013. [2015 January]. Available: http://www.sahlgrenska.se/sv/SU/Forskning/HTA-centrum/Hogerkolumn-undersidor/Hjalpmedel-under-projektet/.

